# Supplementary material for: A Program of Life-Style Modification Improved the Body Weight and Micronutrient Status in Obese Patients after Bariatric Surgery
Source: Nutrients. 2023 Aug 30;15(17):3807. doi: 10.3390/nu15173807 (PMC10490431; doi:10.3390/nu15173807)
Supplement: Supplementary file 1 [file nutrients-15-03807-s001.zip › nutrients-2569543-supplementary.pdf]

Table S1 Utilization of pharmacological treatments (excluding metformin)

	**Total (*n* = 121)**	**Control (*n* = 50)**	**PLM (*n* = 71)**	***p* value**
**ACEi**	25 (20.7 %)	9 (18%)	16 (22.5%)	0.368
**ß-blockers**	6 (5%)	4 (8%)	2 (2.8%)	0.229
**Diuretics**	7 (5.8%)	3 (6%)	4 (5.6%)	1.000
**ARB**	13 (10.7%)	8 (16%)	5 (7%)	0.117
**ACEi/ß-blocker**	7 (5.8%)	3 (6%)	4 (5.6%)	1.000
**Metformin**	16 (13.2 %)	2 (4%)	14 (19.7%)	**0.032**
**Sulfonylureas**	3 (2.5 %)	1 (2%)	2 (2.8%)	1.000
**Insulin + Met/Sulf**	16 (13.2 %)	10 (20%)	6 (8.5%)	0.065
**Statins**	29 (24 %)	16 (32%)	13 (18.3%)	0.082
**Fibrates**	7 (5.8%)	4 (8%)	3 (4.2%)	0.446

Bold, significant.
